# Polysulfides Applied as Formulated Garlic Extract to Protect Tomato Plants against the Root-Knot Nematode *Meloidogyne incognita*

**DOI:** 10.3390/plants10020394

**Published:** 2021-02-18

**Authors:** Reinhard Eder, Erika Consoli, Jürgen Krauss, Paul Dahlin

**Affiliations:** 1 Agroscope, Research Division Plant Protection, Phytopathology and Zoology in Fruit and Vegetable Production, 8820 Wädenswil, Switzerland; reinhard.eder@agroscope.admin.ch (R.E.); erikaconsolli@gmail.com (E.C.); 2 Agroscope, Research Division Plants and Plant Products, Vegetable-Production Extension, 8820 Wädenswil, Switzerland; juergen.krauss@agroscope.admin.ch

**Keywords:** NEMguard^®^ DE, botanical nematicide, plant-parasitic nematodes, soil

## Abstract

The devastating root-knot nematode *Meloidogyne incognita* can cause severe damage to field and greenhouse crops. Due to high economic losses, alternative products are essential to replace banned or strictly regulated nematicides that affect human health and/or the environment. Garlic based products have been previously investigated as environmentally friendly nematicides and their active substances, diallyl polysulfides exist as formulated nematicides on the market. We tested the garlic-based nematicide NEMguard^®^ DE as protective of tomato roots. In vitro evaluation of the lethal concentration (LC) showed strong nematicidal activity with LC_50_ of 0.8 mg/mL after 96 h and LC_90_ of 1.5 mg/mL. NEMguard^®^ DE showed protective effect against *M. incognita* as a single application in small pots and a second application further reduced root galling, significantly. Large greenhouse trials were carried out in two consecutive years to test single and monthly applications of NEMguard^®^ DE. In both years, no controlling effect could be observed on *M.* *incognita*. We assume that the silt content of the loamy sandy soil used had an effect on the polysulfides, inhibiting their nematicidal effect. We conclude that further experiments are necessary to investigate the nematicidal potential of NEMguard^®^ DE under different soil compositions or as a different formulation.

## 1. Introduction

Plant-parasitic root-knot nematodes (RKN) of the genus *Meloidogyne* are severe plant pathogens that cause significant economic losses by inducing the development of root knot galls, altering the plant vascular system and draining essential nutrients from the host [1]. The severe plant damage caused by these obligate biotrophic nematodes can result in wilting, stunted growth, leaf discoloration, root deformation, reduced yield and crop quality (up to the total crop loss). RKN infection can also contribute to reduced plant resistance to other biotic and abiotic stresses and therefore nematode attack is often related to other problems such as drought, nutrient deficiency and different pathogens causing similar diseases, all resulting in economic losses for the growers [1].

*Meloidogyne incognita* is an extremely polyphagous nematode and one of the most common *Meloidogyne* species worldwide, affecting crops in warmer climates and is able to establish in greenhouses of temperate regions, such as Switzerland [1,2].

Chemical and biological nematicides are commonly used against plant-parasitic nematodes with variable efficacy. Although most chemical nematicides show high nematicidal efficacy under field conditions, their impact on human health and on the environment has resulted in enforced regulation or the total ban of the different products [3]. To control plant-parasitic nematodes, grafting, biofumigation, heat treatment, soil compost and crop rotation with poor hosts, catch crops or antagonistic microorganisms have been intensively studied [4,5,6]. However, further management methods, such as plant-based metabolites are being investigated to search for alternative human and environment friendly biopesticides [3,7].

Garlic (*Allium sativum* L.) belongs to the family *Allium*, including other important plants such as onions, leek, shallot, scallion and chives that are cultivated as vegetables, spices and for medicinal purposes [8]. The antimicrobial properties of garlic extracts have already been investigated in the early 19th century [9] and was attributed to the organosulfur compound allicin [10]. Allicin itself is highly unstable and decomposes into various sulfur compounds such as ajoene, dithiines and diallyl polysulfides, including diallyl sulfide, diallyl disulfide, diallyl trisulfide and so on. Up to nine sulfur atoms in the chain have been reported to be present in garlic oil [11,12].

The modes of action and the potential cellular targets of diallyl polysulfides are attributed to the different reactive sulfur species [8]. However, its function is not fully researched in detail. One of its mode of actions is attributed to the metal binding capacity of diallyl polysulfides that can disturb metal homeostasis [8]. In addition, low molecular weight thiols can cause a shift in the redox balance, form different disulfides and alter enzyme activity [8]. Therefore, the formation of reactive oxygen species might cause DNA damage and/or cell apoptosis [13]. However, the primary mode of action of diallyl polysulfides is still uncertain [8] and as for many nematicides, there might be a multi-site inhibitory effect as described for NEMguard^®^ DE containing 45% of garlic extract [14,15]. Despite the uncertain mode of action on plant-parasitic nematodes and other plant pathogens [15], garlic oil, garlic extract, garlic straw and garlic-derived polysulfides have shown to control those pests and pathogens with success [16]. For decades, nematicidal activity of different garlic compositions have been reported against *Meloidogyne* spp. [17,18,19,20] and different garlic-based pesticides are approved/registered and sold to control nematodes and other pests with different levels of success [16].

NEMguard^®^ DE (Certis) is a granular nematicide, produced from allicin extracted from crushed garlic, with polysulfides 3, 4 and 5 as the main active substances [14]. Diatomaceous earth (DE) is used as a carrier of the polysulfides on NEMguard^®^ DE in order to improve soil application and it contains 450 g/kg garlic extract. For its optimal application and in order to allow the release of the polysulfides, 20 mm of irrigation is recommended right after product application in the soil, before planting. For active control, it requires the emulsified state of NEMguard^®^ DE to reach the nematodes in the soil [14]. Its activity against *Meloidogyne* spp. on horticultural crops, such as tomato, carrot, lettuce and melon is reported [21], showing comparable efficacy to synthetic nematicides, in vitro, against the plant-parasitic nematode *Longidorus elongatus* [22,23]. However, other studies have reported only limited nematicidal effects for NEMguard^®^ DE [24].

Therefore, this investigation was carried out to test the direct nematicidal effect of NEMguard^®^ DE in vitro and in a small pot experiment as protective of tomato roots. Furthermore, its nematicidal efficacy against the root-knot nematode *M. incognita* was evaluated in two consecutive greenhouse trials, in 2019 and 2020, when first applied close to the freshly planted roots according to the manufacture and as a monthly treatment around the roots, as novel additional controlling option during the growth season.

## 2. Results and Discussion

### 2.1. In Vitro Effect of Solubelized NEMguard^®^ DE on M. incognita Motility

The in vitro investigation of NEMguard^®^ DE solubilized in tap water revealed increasing nematicidal activity with increasing concentration and prolonged exposure (Table 1). Overall, 0.5 mg/mL or higher concentrations of NEMguard^®^ DE showed significant effect on J2 (immotile) compared to the water control. Only the lowest concentration tested, 0.25 mg/mL did not have a significant impact on the J2, where there was no significantly difference among the number of immotile J2 on the treatment and on the water control. By evaluating the dead J2 with the NaOH method, the number of immotile J2 only significantly differed from the dead ones (NaOH) twice, revealing that the visual observation was in line with the evaluation of the dead J2 (Table 1). Jardim et al. [20] tested the effect of garlic oil on *M. incognita* J2, using the same method to test immotile J2 from dead J2. However, the NaOH method differed from the immotile J2 in the lower concentrations of the oil they tested, showing that there can be differences in the visual assumption, nematode behavior or fitness and therefore the use of NaOH is a good tool for this distinction.

The decline of normal moving J2 with the increasing concentration of NEMguard^®^ DE was also reflected in the gall index (GI) of cucumber roots, essential for evaluating the J2 infectious capacity after being exposed to the nematicide product (Table 1). The GI was significantly reduced at 0.5 mg/mL compared to the water control. However, the strongest reduction of root galling was observed using concentrations between 0.25 mg/mL and 0.5 mg/mL of NEMguard^®^ DE. On these treatments the GI reduced from 6.5, 6.2 and 5.8 to 4.3, 2.6 and 2.4 after 24 h, 48 h and 96 h, of incubation time, respectively. Overall, the highest concentration presented, 2 mg/mL, showed the strongest controlling effect on J2, resulting in no root galls after the J2 were incubated for 48 h and 96 h to NEMguard^®^ DE. These findings are in line with previous investigations, showing that the nematicidal potential of the NEMguard^®^ solution (NEMguard^®^ SC), a different formulation as the one we used for our study against *M. incognita*, is concentration and time dependent [20,26].

**Figure 1:**
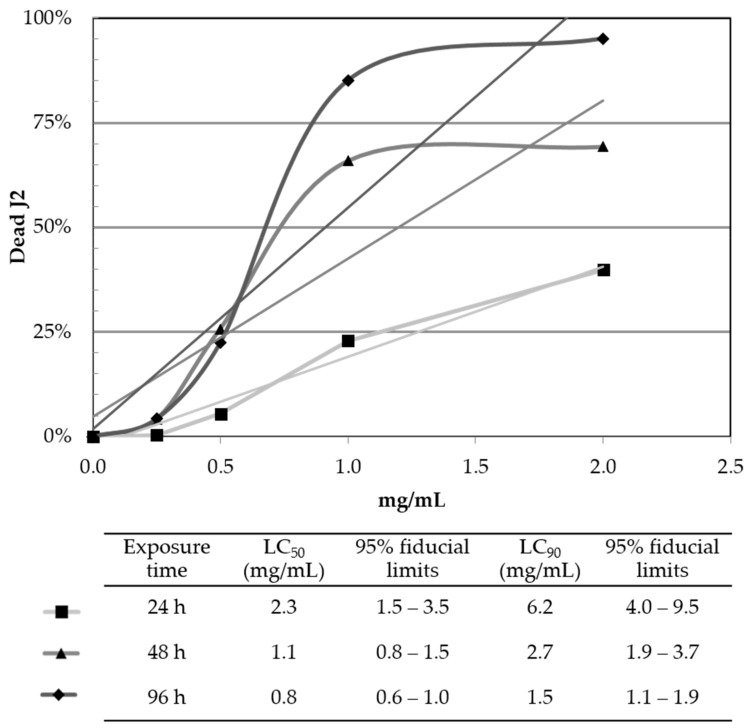
Lethal concentration (LC) LC_50_ and LC_90_ of NEMguard^®^ DE solubilized in water on *Meloidogyne incognita* second stage juveniles (J2) over 24 h, 48 h and 96 h of exposure. Values are means of *n* = 3.

Comparing the immotile J2 among the treatments with 1 and 2 mg/mL, only small changes were recorded, the reduced effect on the J2 can be assumed due to a saturation in water of the NEMguard^®^ DE product. For the calculation of the lethal concentrations (LC), LC_50_ (50%) and LC_90_ (90%), the NaOH data was used (Table 1). The LC_50_ and LC_90_ results supported the assumption that the concentration in water was close before saturation, reaching an LC_90_ of 1.5 mg/mL after 96 h with a 95% fiducial limit ranging between 1.1 to 1.9 mg/mL (Figure 1). Therefore, based on the LC calculations and the gall index (Table 1, Figure 1), 2 mg/mL is sufficient to control over 95% of *M. incognita* J2 in vitro within 96 h. Further investigation (data not shown) using 4 mg/mL NEMguard^®^ DE did not show significant differences compared to 2 mg/mL. As we never reached 100% immotile/dead J2 within 96h, we assume that the progressive insolubility of polysulfides with higher chain length in water [27], reached its saturation point around 2 mg of NEMguard^®^ DE/mL and therefore 4 mg/mL did not differ in the nematicidal effect compared to the 2 mg/mL.

Although, we have not investigated the combination of different polysulfides, it is worthwhile mentioning, that previous studies showed that the lethal concentration of garlic oil or the combination of diallyl disulfide and diallyl trisulfide reached the LC_50_ at lower concentrations than diallyl disulfide or diallyl trisulfide alone [20]. NEMguard^®^ DE mainly consists of polysulfides 3, 4 and 5 and therefore might have a stronger nematicidal effect as the single polysulfide alone.

### 2.2. Efficacy of NEMguard^®^ DE against M. incognita in Small Pots

As soil can have a crucial effect on nematicidal activity [6,14], the controlling effect of NEMguard^®^ DE against *M. incognita* was further tested in small pots using silver sand:soil mixture. Our results revealed that the single application of NEMguard^®^ DE, as recommended by the supplier [14], reduced root galling on tomato plants, 31 days after *M. incognita* inoculation (Table 2). The root galling was even more significantly reduced 59 days after inoculation or when NEMguard^®^ DE was applied a second time as we tested the effect of NEMguard^®^ DE during the period of an additional nematode life cycle. The reapplication of NEMguard^®^ DE was evaluated as an additional investigation and it is not described by the supplier [14]. Furthermore, the treatments, NEMguard 1 and NEMGuard 2 showed reduced numbers of emigrated J2 from the 59 days old root systems compared to the control roots (Table S1).

The height, fresh and dry weights of tomato plants, in addition to root fresh and dry weights and root dry matter did not significantly differ between treatments (31 days after planting), concluding that NEMguard^®^ DE had no phytotoxic effect on the plants (Table 3). In addition, 59 days after planting, the root fresh weight did not differ significantly among treatments and controls (Table S1). These findings are in line with other studies, which report no side effects on plants, when using garlic oil, diallyl disulfide or diallyl trisulfide or garlic essential oil vapor [20,28].

### 2.3. Efficacy of NEMguard^®^ DE against M. incognita under Large-Scale Greenhouse Conditions

**Figure 2:**
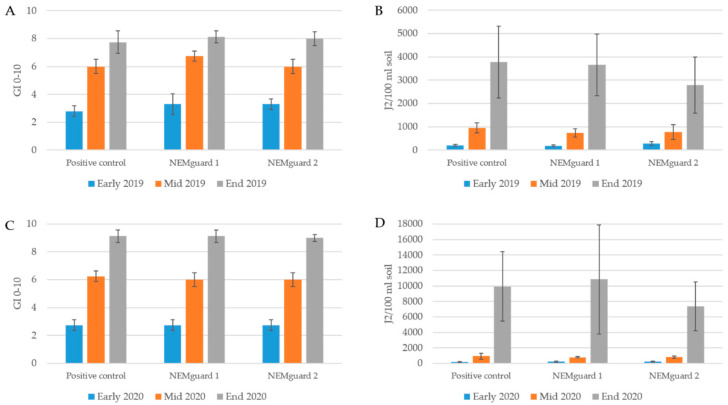
Tomato root gall formation (**A**,**C**) and *Meloidogyne incognita* juvenile second stage (J2) soil population (**B**,**D**) development in the large-scale greenhouse trial over 10 (early), 15 (mid) and 23 (end) weeks testing NEMguard^®^ DE applied before planting (NEMguard 1) and applied before planting followed by a monthly application (NEMguard 2). Suspensions containing approximately 4600 (2019) or 4500 (2020) *M. incognita* eggs/J2 were inoculated per pot. Values for the gall index (GI) according to Zeck [25] and J2 populations are means of *n* = 4 (early and mid) and *n* = 8 (end), respectively. No significant difference according to one-way ANOVA with post-hoc Tukey HSD test among the treatments and the positive control (*p* ≤ 0.05). Positive (p) control = control with *M. incognita*, negative (n) control = soil free of *M. incognita.*.

NEMguard^®^ DE was evaluated in a large-scale tomato trial against *M. incognita,* using the same experimental set up as that of the small pots, over the entire growth seasons in 2019 and 2020 (Figure 2). No significant effect between the two NEMguard^®^ DE treatments and the nematode control was observed on early, mid and late root gall index in both years (Figure 2A,C). However, despite no significant differences, the repeated NEMguard^®^ DE application had a reduced J2 population at the end of both growth seasons (Figure 2B,D), indicating that there is an effect on the J2 in the soil. However, as the nematicidal effect of NEMguard^®^ DE increases over time and as the plant roots in the pots had a limited area for growth, freshly hatched J2 might have an advantage in the well-rooted environment to escape from the nematicidal polysulfides applied monthly. Therefore, the reducing effect on the *M. incognita* soil population was not sufficient to reduce root galling. Similar results were reported with reduced J2 population in soil treated with NEMguard^®^ DE and a slight reduced galling on tomato roots during a greenhouse trail in north Germany [24]. On the other hand, Ladurner et al. [21] reported sufficient control by NEMguard^®^ granules on tomato, carrot, lettuce and melon, and Peçen et al. [29] reported a root gall reduction of 9.62% compared to the control. 

As in the small pot experiment, no phytotoxic effects were visible on the plants within the different treatments. The tomato yield in 2019 and 2020 differed at the end of the growth season, however only the control plants (free of nematodes), with the highest tomato yield were significantly different to the other plants (Table S2). Comparing the tomato yield over time, the different treatments and controls showed that the nematode free control differed from the infected plants, between mid to end of the growth seasons, resulting in a higher accumulated yield in both years (Figure S1A,B). These results show the impact of *M. incognita* on the final yield and the impact they can cause on the growers economic success. *M. incognita* requires 400 °C-days/life cycle [30]. Thus, based on the accumulated temperature at the greenhouse, in 2019 and 2020 trials 4.8 live cycles (1921 °C-days) and 5.0 life cycles (2011 °C-days) were completed. In this way, the increasing yield loss can be attributed to the increase of *M. incognita* population over time.

One factor contributing to the lack of control of *M. incognita* in the large greenhouse trial could be the composition of the soil. The soil used in the pot experiments was characterized as sandy loam, which is described to have the lowest restriction for diallyl disulfide movement compared to fine sand or silty clay loam [31]. However, the soil used had a silt contend of 29.2%, which might have caused an effect on NEMguard^®^ DE, as silt is described to inhibit the polysulfide activity [31]. Therefore, the suppliers do not recommend NEMguard^®^ DE in silty soils (Best practice application guide [14]). Presumably, the lack of *M. incognita* control might be based on the content of silt present in the soil used on our experiment and the possibility, that the well-rooted pots gave the hatched J2 the possibility to find a host root with no or little exposure to the reapplied NEMguard^®^ DE. In this way, additional investigations are needed to reveal to which extend soil texture can affect the efficacy of NEMguard^®^ DE, since nematicidal activity was observed at the in vitro assay and at the small pot experiment, where one part of soil was supplemented with 2 parts of silver sand.

Therefore, further studies on determining maximum silt content in the soil and the influence of different formulations on the efficiency of the active ingredients (such as NEMguard^®^ SC) are needed. This will allow to give the farmers additional support when choosing to use NEMguard^®^ DE or other RKN management strategies, as the silt contend in the Swiss top soil layer can vary between 6% and 73% [32].

## 3. Materials and Methods

### 3.1. Preperation of Nematode Inoculum

*Meloidogyne incognita* (Mi-virulent population described by Hallmann and Kiewnick [2]) were cultured under greenhouse conditions on *Solanum lycopersicum* cv. Oskar (60% humidity, 25 °C/19 °C, 15 h/9 h day-night cycle). Heavily galled root systems were placed under the mist chamber at 23 °C to extract second stage juveniles (J2) [4]. *M. incognita* eggs were extracted from 1 cm cut roots submerged in 1% NaClO water solution and shaken for 3 min in a 0.5 L plastic bottle. A 20 μm mesh sieve was used to collect eggs and to rinse thoroughly with water to remove excessive NaClO. Collected eggs were rinsed on an Oostenbrink dish and left at room temperature for hatching. *M. incognita* eggs and/or J2 were stored at 6 °C for a maximum of 5 days before use.

### 3.2. In Vitro Effect of NEMguard DE on Second Stage Juvenile Meloidogyne incognita Motility

The NEMguard^®^ DE inhibitory effect on *M. incognita* J2 was tested in vitro in an aqueous assay. NEMguard^®^ DE was prepared as a double strength suspension by re-suspending granules in tap water overnight. For each treatment and time point, 1550 J2 were exposed to final concentrations of 0.25, 0.5, 1, 2 or 4 mg/mL of NEMguard^®^ DE. After 24 h, 48 h and 96 h, we examined three times 100 J2 for each treatment, using a light microscope (40x magnification) to monitor the mortality (normal motility, affected motility and immotile (elongated)) (*n* = 3). To determine live immotile (elongated) J2, the NaOH method described by Chen and Dickson [33] was applied and the triplicate (*n* = 3) of the same 100 J2 was monitored according to their mortality. For each treatment and time point, five times 250 J2 were washed with tap water free from NEMguard^®^ DE, using a 20 μm mesh sieve. Each of the washed 250 J2 were applied to a pre-germinated cucumber seedling to evaluate their infection capacity in a bio test. Cucumber seedlings were grown in 30 mL pots (*n* = 5) filled with a soil:silver sand mix (1:3, *v*/*v*) and were placed in a growth chamber set to 23 °C ± 2 °C, 16:8 day:night photoperiod and 60% relative humidity. Four weeks after inoculation, cucumber roots were washed free of soil and *M. incognita* root gall formation was determined on a scale from 0-10 according to Zeck [25].

### 3.3. Small-Scale Greenhouse Test of NEMguard^®^ DE against Meloidogyne incognita

Small greenhouse experiments were conducted in pots (∅ = 13 cm) filled with soil:silver sand mix (1:2 *v*/*v*), inoculated with approximately 2000 J2/pot, covered to keep soil moist and let stand for 3 days. The experiment was split into a negative control, a positive control with nematodes, a single NEMguard^®^ DE treatment (NEMguard 1) and a double, NEMguard^®^ DE + NEMguard^®^ DE treatment (NEMguard 2), with the second treatment being applied during plant growth. Each treatment was replicated six times (*n* = 6). “Root balls” of 5-week-old tomato plants cv. Oskar were embedded in 1 gram NEMguad^®^ DE/plant, by well distributing the granules around the “root ball” and planting into the pots. The second NEMguard^®^ DE application was carried out 24 days after planting, by preparing a 1 g NEMguard^®^ DE tap water suspension as a 25 mL solution, directly applied at the soil surface of each plant. Plants were grown for 31 days and 59 days at 22 °C ± 2 °C, with a 16:8 day:night photoperiod and watered according to the plant needs. For each pot and time point, the gall index was determined after Zeck [25], on a scale from 0–10 (with 0 being a healthy uninfected plant root system and 10 a root-knot nematode destroyed plant root system). Plant height, fresh and dry weights, root fresh and dry weights and root dry matter, were determined 31 days after treatment. Root weight and J2 hatched from root systems placed under the mist chamber were taken at 59 days after treatment.

### 3.4. Nematical Effect of NEMguard^®^ DE against Meloidogyne incognita under a Large-Scale Tomato Trial 

The large-scale greenhouse tomato trial took place at Agroscope in Wädenswil, using 64 x 15 L pots. The experiment was designed as four rows with treatments of NEMguard^®^ DE (NEMguard 1), NEMguard^®^ DE + NEMguard^®^ DE (NEMguard 2), positive and negative nematode controls randomly arranged as pairs of two. Pots were filled to ⅔ with steamed field soil characterized as sandy loam (containing humus 3.2% *w*/*w*; clay 16.7% *w*/*w*; silt 29.2% *w*/*w*) and inoculated with a suspension containing approximately 4600 and 4500 *M. incognita* eggs (76%/55%) and J2 (24%/45%) per pot, in 2019 and 2020, respectively. NEMguard^®^ DE was applied 3 days prior to planting, by mixing 1 g NEMguard^®^ DE/1 L of soil into the last 5 L of soil/pot. Controls were treated accordingly without NEMguard^®^ DE. All pots were watered immediately as recommended from the supplier [14]. For the NEMguard^®^ DE + NEMguard^®^ DE treatment, every 4 weeks NEMguard^®^ DE was solubilized in tap water and applied as 5 g in 50 mL/plant and rinsed with 50 mL of tap water.

The grafted tomato Roterno F1 RZ and the Mi-resistant rootstock Maxiford (72-230; Rijk Zwaan) were used in both trials. Plants were watered and fertilized with water-soluble NPK fertilizer (Kristalon Red Acid, Yara, UK) according to the plant growth stage. In 2019 and 2020, root galling was indexed at week 10 (early (*n* = 4)), 15 (mid (*n* = 4)) and 23 (end (*n* = 8)). For each root rating, *M. incognita* soil population was determined in 100 ml soil, using the Oostenbrink dish method [4]. Extracted J2 were determined under the light microscope at 40x magnification.

The accumulated greenhouse temperature sum was recorded every two minutes during both trials, growth seasons 2019 and 2020 and the number of *M. incognita* life cycles were determined according to Ornat and Sorribas [30].

## 4. Conclusions

As a botanical nematicide, NEMguard^®^ DE appears to have advantages as biological available product and potentially supporting organic farmers on the control of plant parasitic nematodes. However, according to our investigations, the nematicidal activity observed in vitro, did not remain in the large greenhouse experiment and insufficiently controlled *Meloidogyne incognita*. Nevertheless, nematicidal effects were observed in our small pot experiment where the soil was supplemented with silver sand. We conclude that, further testing of the nematicidal potential of NEMguard^®^ DE under different soil compositions is essential, in order to allow drawing conclusions if the soil composition has an impact on the nematicidal properties of NEMguard^®^ DE. In addition, different NEMguard^®^ formulations, such as NEMguard^®^ SC might enhance the nematicidal effect of the garlic-based products under different soil compositions and should be further investigated.

## Figures and Tables

**Table 1:** In vitro effect of NEMguard^®^ DE on *Meloidogyne incognita* second stage juveniles (J2).

NEMguard^®^ DE		Nematode Movement (%)			NaOH	Gall Index
Exposure Time	Concentration mg/mL	N (%)	A (%)	I (%)	D (%)	
24 h	0 (control)	100 a	0.0 a	0.0 a	0	6.2 a
	0.25	90.7 b	9.0 b	0.3 a	0	6.5 a
	0.5	1.9 c	89.6 c	8.5 c	15	4.3 b
	1	0.2 c	72.5 d	27.3 d	23	0.8 c
	2	0.2 c	66.5 d	33.3 d	39.8	0.4 c
48 h	0 (control)	100 a	0.0 a	0.0 a	0	6.4 a
	0.25	65.1 b	34.5 b	0.4 a	0	6.2 a
	0.5	0 c	85.8 c	14.2 b	26	2.6 b
	1	0.0 c	39.7 b	60.3 c	65.9	0.0 c
	2	0.0 c	38.2 b	61.8 c	69.3 *	0.0 c
96 h	0 (control)	98.4 a	1.4 a	0.2 a	0	6 a
	0.25	74.9 b	24.5 b	0.6 a	0	5.8 a
	0.5	0.0 c	71 c	28.2 b	22.5	2.4 b
	1	0.0 c	6.1 a	93.9 c	85.1 *	1.2 bc
	2	0.0 c	3.6 a	96.4 c	95.1	0.0 c

Percentage (%) of normal (N), affected (A), immotile (I) and dead (D) J2 (*n* = 3). NEMguard^®^ DE exposed J2 were inoculated on germinated *Cucumis sativus* seedlings and three weeks later, root gall indexing (GI) was determined according to Zeck [25] (*n* = 5). Means within the same column and exposure times followed by the same letter are not significantly different according to one-way ANOVA with post-hoc Tukey HSD test. *p* < 0.05. * show significant differences between the row I and D, calculated using a *t*-test (*n* = 3) *p* < 0.05.

**Table 2:** Root gall indexing (GI) of tomato plants grown in small pots for 31 and 59 days after planting (DAP) to evaluate the nematicidal effect of NEMguard^®^ DE against *Meloidogyne incognita*.

Treatment	GI 31 DAP	GI 59 DAP
pControl	5.7 ± 0.8 a	7.3 ± 0.5 a
NEMguard 1	4.8 ± 0.8 ab	6.3 ± 0.5 b
NEMguard 2	4.2 ± 0.8 b	5.5 ± 0.6 c

Root gall indexing (GI) was determined according to Zeck [25]. Values are means of *n* = 6, same letter in the same column indicate no significant differences using a one-way ANOVA (post-hoc Tukey HSD test). NEMguard 1 = NEMguard^®^ DE applied before planting; NEMguard 2 = NEMguard^®^ DE applied before planting followed by a repeated application 24 days after planting. Positive (p) control = control with *M. incognita*, negative (n) control = soil free of *M. incognita*.

**Table 3:** Height, fresh and dry weights of tomato plants 31 days after planting into *Meloidogyne incognita* infested soil treated with NEMguard^®^ DE.

Title 1	Plant Height (cm)	Plant Fresh Weight (g)	Plant Dry Weight (g)	Root Fresh Weight (g)	Root Dry Weight (g)	Root Dry Matter (%)
nControl	79.0 ± 11.6 a	31.7 ± 7.0 a	4.0 ± 1.2 a	10.3 ± 3.1 a	0.9 ± 0.2 a	9.3 ± 3.3 a
pControl	72.5 ± 9.0 a	31.3 ± 7.6 a	3.8 ± 1.4 a	10.4 ± 1.9 a	0.9 ± 0.2 a	8.8 ± 1.1 a
NEMguard 1	75.0 ± 5.7 a	32.7 ± 2.3 a	4.3 ± 1.0 a	9.5 ± 1.3 a	0.9 ± 0.1 a	9.7 ± 1.5 a
NEMguard 2	77.5 ± 8.2 a	33.5 ± 6.0 a	4.7 ± 1.5 a	10.5 ± 1.9 a	1.1 ± 0.3 a	10.8 ± 2.2 a

Values are means of *n* = 6, same letter in the same column indicate no significant differences using a one-way ANOVA (post-hoc Tukey HSD test). NEMguard 1 = NEMguard^®^ DE applied before planting; NEMguard 2 = NEMguard^®^ DE applied before planting followed by a repeated application 24 days after planting. Positive (p) control = control with *M. incognita*, negative (n) control = soil free of *M. incognita*.

## Data Availability

Data is contained within the article or supplementary material.

## Supplementary Materials

The following are available online at https://www.mdpi.com/2223-7747/10/2/394/s1. Figure S1: Accumulated tomato yield over time from *Meloidogyne incognita* infested plants after treatment with NEMguard^®^ DE from two consecutive years (A = 2019 and B = 2020). Table S1: Tomato plants root weight and second stage juveniles hatched from roots grown for 59 days in *Meloidogyne incognita* infected soil treated with NEMguard^®^ DE as single (NEMguard 1) and repeated application (NEMguard 2). Table S2: Tomato yields from *Meloidogyne incognita* infested plants after treatment with NEMguard^®^ DE from two consecutive years.
