# Supplementary material for: Polysulfides Applied as Formulated Garlic Extract to Protect Tomato Plants against the Root-Knot Nematode Meloidogyne incognita
Source: Plants (Basel). 2021 Feb 18;10(2):394. doi: 10.3390/plants10020394 (PMC7922410; doi:10.3390/plants10020394)
Supplement: Supplementary file 1 [file plants-10-00394-s001.pdf]

Supplementary Materials

Supplementary Tables

Table S1. Tomato plants root weight and second stage juveniles (J2) hatched from roots grown for 59 days in *Meloidogyne incognita* infected soil treated with NEMguard® DE as single (NEMguard 1) and repeated application (NEMguard 2).

Treatment	Root fresh weight (g)	J2 per plant
nControl	20.1 ± 2.0a	0
pControl	23.9 ± 4.0a	128777.8 ± 31106.0a
NEMguard 1	19.9 ± 2.8a	113138.9 ± 56040.6a
NEMguard 2	23.4 ± 4.8a	76805.6 ± 60023.2a

Values are means of n = 6, same letter in the same column indicate no significant differences using a one-way ANOVA (post-hoc Tukey HSD test). NEMguard 1 = NEMguard® DE applied before plant; NEMguard 2 = NEMguard® DE applied before plant followed by a repeated application 24 days after planting. Positive (p) control = control inoculated with *M. incognita*. Negative (n) control = soil free of *M. incognita.*

Table S2. Tomato yields from *Meloidogyne incognita* infested plants after treatment with NEMguard® DE from two consecutive years.

Growth season	Treatments	Average weight g/harvest and plant	Average no. of fruits /harvest and plant	Average fruit weight g/harvest and plant	Total fruit weight per plant (kg)	Yield potential (%)
2019	nControl	1241.5 ± 555.9a	16.6 ± 6.8a	74.8 ± 10.6a	14.9 ± 1.4a	100
	pControl	1024.8 ± 514.8b	15.9 ± 6.8a	62.7 ± 13.2b	12.3 ± 0.6b	82.5
	NEMguard 1	1037.0 ± 551.7b	15.6 ± 6.8a	61.6 ± 14.8b	12.4 ± 1.0b	83.5
	NEMguard 2	990.5 ± 554.2b	15.1 ± 6.5a	60.4 ± 16.3b	11.9 ± 1.3b	79.8
2020	nControl	1420.4 ± 500.9a	19.7 ± 5.8a	72.1 ± 12.5a	17.0 ± 1.3a	100
	pControl	1199.7 ± 470.5b	18.2 ± 5.2a	64.7 ± 14.1a	14.4 ± 1.8b	84.5
	NEMguard 1	1158.0 ± 510.6b	17.2 ± 5.9a	66.0 ± 16.8a	13.9 ± 0.9b	81.5
	NEMguard 2	1268.1 ± 508.8ab	19.2 ± 5.7a	65.8 ± 16.9a	15.2 ± 0.9ab	89.3

Values are means/sums of n = 8. Same letter in the same column indicates no significant differences using a one-way ANOVA (post-hoc Tukey HSD test). NEMguard 1 = NEMguard® DE applied before planting; NEMguard 2 = NEMguard® DE applied before planting followed by a repeated application (monthly) after planting. Positive (p) control = control inoculated with *M. incognita*, negative (n) control = soil free of *M. incognita.*

Supplementary Figures

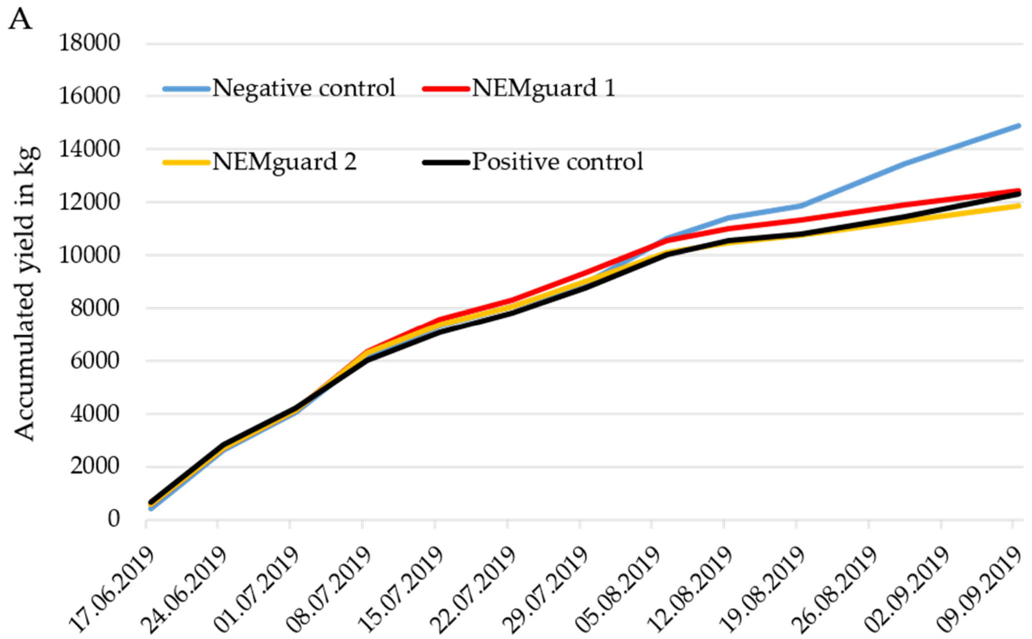

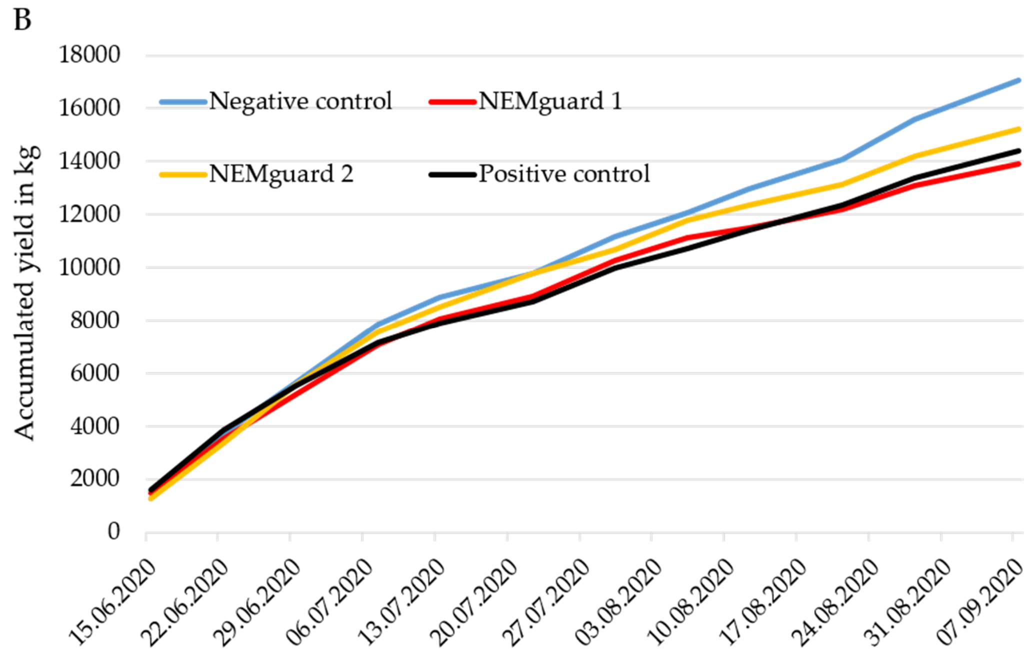

Figure S1. Accumulated tomato yield over time from *Meloidogyne incognita* infested plants after treatment with NEMguard® DE from two consecutive years (A=2019 and B=2020). Positive control = control inoculated with *M. incognita*, negative control = soil free of *M. incognita*. NEMguard 1 = NEMguard® DE applied before planting; NEMguard 2 = NEMguard® DE applied before planting followed by repeated monthly applications.
